# The real-world safety of atezolizumab as second-line or later treatment in Japanese patients with non-small-cell lung cancer: a post-marketing surveillance study

**DOI:** 10.1093/jjco/hyac024

**Published:** 2022-03-23

**Authors:** Yuichiro Ohe, Naoya Yamazaki, Nobuyuki Yamamoto, Haruyasu Murakami, Kiyotaka Yoh, Shigehisa Kitano, Hideyuki Hashimoto, Ayako Murayama, Sayuri Nakane, Akihiko Gemma

**Affiliations:** Department of Thoracic Oncology, National Cancer Center Hospital, Tokyo, Japan; Department of Dermatologic Oncology, National Cancer Center Hospital, Tokyo, Japan; Internal Medicine III, Wakayama Medical University, Wakayama, Japan; Division of Thoracic Oncology, Shizuoka Cancer Center, Sunto-gun, Japan; Department of Thoracic Oncology, National Cancer Center Hospital East, Kashiwa, Japan; Division of Cancer Immunotherapy Development, Advanced Medical Development Center, The Cancer Institute Hospital of the Japanese Foundation for Cancer Research, Tokyo, Japan; Real World Data Science Department, Drug Safety Division, Chugai Pharmaceutical Co., Ltd., Tokyo, Japan; Real World Data Science Department, Drug Safety Division, Chugai Pharmaceutical Co., Ltd., Tokyo, Japan; Real World Data Science Department, Drug Safety Division, Chugai Pharmaceutical Co., Ltd., Tokyo, Japan; Department of Pulmonary Medicine and Oncology, Graduate School of Medicine, Nippon Medical School, Tokyo, Japan

**Keywords:** atezolizumab, non-small-cell lung cancer, immune-related reactions, interstitial lung disease, safety

## Abstract

**Background:**

We conducted a post-marketing surveillance study to evaluate the clinical tolerability and safety of atezolizumab in Japanese patients with non-small-cell lung cancer (NSCLC).

**Methods:**

This prospective, observational post-marketing cohort study was conducted in NSCLC patients who received atezolizumab 1200 mg every 3 weeks at 770 facilities in Japan between April 18, 2018, and March 31, 2020 (study number UMIN000031978). Case report forms were completed, recording patient characteristics, treatment details, adverse events, adverse drug reactions (ADRs), their severity, onset and outcomes. Follow-up was for 12 months or until atezolizumab discontinuation.

**Results:**

Overall, 2570 patients were included, median age was 69.0 years, and 69.9% were males. ADRs were reported in 29.1% of patients, most commonly pyrexia (4.2%). Grade ≥ 3 ADRs occurred in 9.7% of patients aged <75 and 9.7% of those aged ≥75 years. The incidence of Grade ≥ 3 ADRs was not affected by the number of lines of previous treatment or the presence or history of an autoimmune disorder. Immune-related ADRs of interest that occurred in >1% of patients were interstitial lung disease (ILD; 4.4%), endocrine disorder (4.3%), and hepatic dysfunction (2.8%). ILD was significantly more common in patients with a history of, or concurrent, ILD versus those without (*P* ≤ 0.001). Risk factors of Grade ≥ 3 ADRs were a history of, or concurrent, ILD. Grade 5 ADRs occurred in 35 patients, 11 of whom had concurrent ILD.

**Conclusions:**

This large cohort study confirmed the clinical tolerability of atezolizumab in a real-world group of Japanese patients with NSCLC.

## Teaser

This large-scale (*n* = 2570) post-marketing surveillance study confirmed the excellent clinical tolerability and safety of atezolizumab in a real-world cohort of real-world Japanese patients with non-small cell lung cancer.

## Introduction

Lung cancer is the leading cause of cancer-related mortality in Japan, with 53 200 deaths in men and 22 300 deaths in women in 2020 (1). In Japan, lung cancer causes 24% of cancer-related deaths in men and 14% in women (1), indicating that there is a high unmet medical need to reduce lung cancer mortality. This is particularly true for non-small cell lung cancer (NSCLC), which accounts for ~80% of lung cancer diagnoses in Japan (2).

Standard chemotherapy, which has long been the mainstay of treatment for NSCLC, has limited efficacy, and only 30%–60% of patients are still alive 2 years after treatment (3–5). Immunotherapy is emerging as a promising option to improve outcomes in patients with NSCLC, including agents that target programmed cell death protein-1 (PD-1) and programmed cell death ligand-1 (PD-L1) (6).

Atezolizumab is a humanized anti-PD-L1 antibody that inhibits the binding of PD-L1 to PD-1, and the subsequent signalling that leads to activation of antitumour immunity (7). In addition, it has been reported that PD-L1 inhibitors have less effect on PD-L2 compared with PD-1 antibodies (8), and therefore are associated with a lower incidence of pneumonitis than the PD-1 antibodies are (9). Atezolizumab was approved in Japan based on the results of the global phase III OAK study (10). In the OAK study, atezolizumab significantly prolonged median overall survival (OS) by approximately 4.2 months compared with docetaxel in previously treated patients with metastatic NSCLC, and was better tolerated, with a lower overall incidence of adverse events (AEs) (10). Approximately 21% of patients in the OAK study were Asian.

Although the efficacy of immunotherapy has been demonstrated in global clinical trials, most of the patients participating in these trials were Caucasian (white) and Asian patients were under-represented in those studies (11). In addition, there may be differences in the frequency of epidermal growth factor receptor (EGFR) mutations and hepatitis B virus (HBV) carriage between Asian and Caucasian patients that could affect the emergence of immune-related events, which would not be apparent in the global clinical studies (12).

In addition to these concerns, the strict inclusion criteria of phase III studies limits their external validity; therefore, investigations in clinical practice are considered essential to establish safety and effectiveness in the heterogeneous of patients who are seen in clinical practice (13,14). This is especially true for the subgroups of patients who are often excluded from clinical trials, including those aged ≥70 years who make up ~50% of lung cancer patients in Japan (2), and patients with respiratory conditions, including interstitial lung disease (ILD).

Therefore, the regulatory approval of atezolizumab in Japan required that an all-case post-marketing surveillance study be conducted to evaluate the clinical tolerability and safety of atezolizumab in Japanese patients with NSCLC. Herein, we describe that study.

## Methods

This was a prospective, observational post-marketing surveillance study conducted in patients with unresectable, progressive or recurrent NSCLC who were scheduled to receive atezolizumab monotherapy at 770 facilities in Japan. Data management and analysis were undertaken by a contract research organization on behalf of Chugai Pharmaceuticals, Ltd. Eligible patients were registered using a central registration system between April 18, 2018, and September 30, 2018. At the end of the enrollment period, all patients who received atezolizumab at the participating institution were confirmed to be enrolled.

This study was registered as UMIN000031978 on the clinical trial registration site before the start of the study. The study was conducted in accordance with the Good Post-marketing Study Practice (GPSP) of the Ministry of Health, Labor, and Welfare of Japan approved by the Japanese authorities. Under these regulations, central Institutional Review Board (IRB) oversight and patient consent are not required.

Data were recorded in case report forms (CRFs) for each patient, including the patient’s clinical and demographic characteristics; tumour type; medical history and prior cancer treatments; atezolizumab treatment details; other cancer treatments (e.g. radiotherapy); and AEs. Atezolizumab was administered at a dose of 1200 mg every 3 weeks. Follow-up was for 12 months or until discontinuation of atezolizumab, and included data collected between April 18, 2018, and March 31, 2020. Data on all adverse drug reactions (ADRs; i.e. AEs considered to be at least possibly related to atezolizumab) and ADRs of interest were recorded, including the timing of their onset, severity, and outcome. The ADRs of interest were ILD, hepatic dysfunction, colitis/severe diarrhoea, pancreatitis, type 1 diabetes mellitus, endocrine disorder, encephalitis/meningitis, neurologic toxicity, myasthenia gravis, severe skin disorder, renal dysfunction, myositis/rhabdomyolysis, myocarditis, hemolytic anaemia, immune thrombocytopenic purpura (ITP), and infusion reactions. AEs and ADRs were classified using the Medical Dictionary of Regulatory Affairs (MedDRA) version 22.1 system organ class (SOC) and preferred term (PT). The severity of each AE was graded according to the Common Terminology Criteria of Adverse Events (CTCAE) v 4.0-JCOG classification. No information on effectiveness was collected.

### Statistical analysis

A target sample size of 1000 cases was determined to have 80% power to detect ADRs that occurred in ≥1 patient in the phase III OAK study (10) and to detect ≥10 cases of ILD, hepatic dysfunction, endocrine disorder, neuropathy, severe skin disorder, and renal dysfunction. Even if the number of registered patients exceeded 1000, registration was continued until the Japanese regulatory authority considered that sufficient safety data had been collected.

The incidences of all ADRs and ADRs of interest and their 95% confidence intervals were calculated, as were the time to onset, treatment and outcome (e.g. recovery, remission) for all ADRs and ADRs of interest. The incidence rate of ILD was also calculated in patient subgroups based on the history of ILD and presence/absence of concurrent ILD at baseline, and compared between subgroups using the Pearson’s χ^2^ test. Missing data were not imputed.

The IRB of one facility declined to allow patient data from that hospital to be included, because they disallowed publication of patient-related data. Therefore, the full analysis set includes all patients who received atezolizumab at any of the other 769 participating hospitals.

Data analysis was undertaken using Statistical Analysis System (SAS) version 9.2.

## Results

### Patient disposition and characteristics

**Figure 1:**
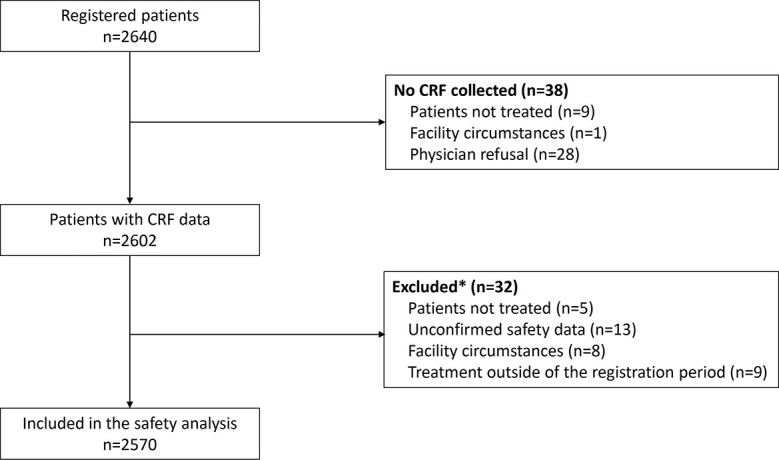
Patient disposition. *Patients could have more than one reason for exclusion. CRF, case report form.

Of the 770 eligible institutions, 685 participated and CRFs from 684 institutions are included in this analysis. Although the target sample size was 1000 patients, 2640 patients were registered and data were available from 2602 patients. Data were not collected from 38 patients for the following reasons: the patient did not receive atezolizumab (*n* = 9), the facility closed the participating department (*n* = 1), and physician refusal (*n* = 28). Data from 32 patients were excluded, including eight patients from the institution that declined to participate in the analysis, 13 patients with unconfirmed safety data, nine patients who were treated outside of the registration period, and five patients who did not receive atezolizumab. Therefore, the final analysis included 2570 patients (Figure 1).

The characteristics of the 2570 patients included in the analysis are shown in Table 1. Overall, 1797 patients (69.9%) were men and 773 patients (30.1%) were women. Patients were aged between 30 and 90 years (median 69.0 years), with 658 patients (25.6%) aged ≥75 years. The baseline Eastern Cooperative Oncology Group (ECOG) performance status was ≤1 in 2099 patients (81.7%) and ≥2 in 465 patients (18.1%). Eighty-seven patients (3.4%) had a history of ILD, and ILD was present at the time of initiating atezolizumab in 138 patients (5.4%). Twenty-seven patients (1.1%) had a medical history of autoimmune disorders, and 98 patients (3.8%) were experiencing an autoimmune disorder when they began atezolizumab (Table 1).

**Table 1:** Patient baseline demographics and clinical characteristics

	Atezolizumab (*n* = 2570)
Sex	
Male	1797 (69.9)
Female	773 (30.1)
Age, years, median (range)	69.0 (30–90)
Age categories	
<75 years	1912 (74.4)
≥75 years	658 (25.6)
Tumour histology	
Adenocarcinoma	1920 (74.7)
Squamous cell carcinoma	440 (17.1)
Large cell carcinoma	40 (1.6)
Other	121 (4.7)
Unknown or data missing	49 (1.9)
ECOG performance status	
≤1	2099 (81.7)
2	387 (15.1)
3	72 (2.8)
4	6 (0.2)
Unknown or data missing	6 (0.2)
Line of atezolizumab therapy	
Second	919 (35.8)
Third	552 (21.5)
Fourth or later	1018 (39.6)
Other	77 (3.0)
Unknown or data missing	4 (0.2)
Medical history	
Interstitial lung disease	87 (3.4)^a^
Autoimmune disease	27 (1.1)^b^
Concurrent complications	
Interstitial lung disease	138 (5.4)^c^
Autoimmune disease	98 (3.8)^b^
Medical history of, or concurrent, HBV infection	27 (1.1)
Previous immunotherapy	826 (32.1)^a^
Nivolumab	610 (23.7)
Pembrolizumab	252 (9.8)
Atezolizumab^d^	59 (2.3)
Other	3 (0.1)
Treatment within previous 3 months	
Chemotherapy	1767 (68.8)^a^
EGFR tyrosine kinase inhibitors	127 (4.9)^a^
Previous radiotherapy	368 (14.3)^a^
Brain	150 (5.8)
Bone	129 (5.0)
Lungs including lymph nodes	76 (3.0)
Other	47 (1.8)

All data are *n* (%) unless otherwise stated.

^a^Data were missing for five patients.

^b^Data were missing for seven patients.

^c^Data were missing for six patients.

^d^Patients in this group had participated in clinical trials with atezolizumab or had transferred from another hospital where they had received atezolizumab.

ECOG, Eastern Cooperative Oncology Group; EGFR, epidermal growth factor receptor; HBV, hepatitis B virus; SD, standard deviation.

### Previous treatments and atezolizumab treatment

Overall, 919 patients (35.8%) were receiving atezolizumab as second-line treatment, 552 patients (21.5%) as third-line, and 1018 patients (39.6%) as fourth-line or later. Prior treatment included cancer immunotherapy in 826 patients (32.1%) and anticancer drugs other than cancer immunotherapy in 1767 patients (68.8%). Patients received between 1 and 24 (median 3.0) doses of atezolizumab, and the treatment duration ranged from 1 day to 565 days (median 62.0 days). The main reasons for discontinuing atezolizumab were: disease progression (*n* = 1684; 65.5%), AEs (*n* = 273; 10.6%) and cancer-related death (*n* = 269; 10.5%; Table 1).

### Adverse drug reactions

A total of 1171 ADRs developed in 748 patients (incidence 29.1%). Grade ≥ 3 ADRs developed in 250 patients (9.7%; Table 2). The incidence of Grade ≥ 3 ADRs was the same in patients aged <75 years (*n* = 186/1912; 9.7%) and in those aged ≥75 years (*n* = 64/658; 9.7%). Grade ≥ 3 ADRs were reported in 114 of the 919 patients (12.4%) receiving atezolizumab as second-line treatment, 58/552 of patients (10.5%) receiving it third-line and 73/1018 of patients (7.2%) receiving fourth-line or later atezolizumab. The incidence of Grade ≥ 3 ADRs was 7.4% in patients with a history of autoimmune disease (*n* = 2/27) and 9.8% in those without an autoimmune disease history (*n* = 248/2536), and in 8.2% (*n* = 8/98) and 9.8% (*n* = 241/2465) in patients with and without concurrent autoimmune disease, respectively. The incidence of Grade ≥ 3 ADRs was 18.4% in patients with a history of ILD (*n* = 16/87) and 9.4% in those without ILD history (*n* = 234/2478; *P* = 0.0056), and in 24.6% (*n* = 34/138) and 8.9% (*n* = 215/2426) in patients with and without ILD (*P* < 0.0001), respectively. Grade ≥ 3 ADRs were reported in 9.6% (*n* = 238/2486) of patients with an ECOG performance status of ≤2 and in 14.1% (*n* = 11/78) of those with a performance status of ≥3. Risk factors of Grade ≥ 3 ADRs were a history of, or concurrent, ILD (Table 2). The incidence of Grade ≥ 3 ADRs also tended to be higher in patients with a poorer performance status (ECOG 3–4 vs. ≤2) (Table 2).

**Table 2:** Incidence of any adverse drug reactions (ADRs) or Grade ≥ 3 ADRs in patient subgroups

Patient group	*n*	Any ADRs		Grade ≥ 3 ADRs	
		No. of patients with ADRs (%)	No. of ADR events	No. of patients with ADRs (%)	No. of ADR events
All patients	2570	748 (29.1)	1171	250 (9.7)	316
Sex					
Male	1797	547 (30.4)	827	176 (9.8)	222
Female	773	201 (26.0)	344	74 (9.6)	94
Age categories					
<75 years	1912	570 (29.8)	883	186 (9.7)	231
≥75 years	658	178 (27.0)	288	64 (9.7)	85
Tumour histology					
Adenocarcinoma	1920	547 (28.5)	870	184 (9.6)	232
Squamous cell carcinoma	440	137 (31.1)	198	46 (10.5)	56
Large cell carcinoma	40	14 (33.3)	17	4 (10.0)	4
Other	121	34 (28.1)	61	12 (9.9)	18
Unknown or data missing	49	16 (32.7)	24	4 (8.2)	6
ECOG performance status					
≤1	2099	635 (30.3)	998	200 (9.5)	260
2	387	90 (23.2)	144	38 (9.8)	43
3	72	16 (22.2)	20	11 (15.3)	12
4	6	2 (33.3)	2	0	0
Unknown or data missing	6	5 (83.3)	7	1 (16.7)	1
Line of atezolizumab therapy					
Second	919	327 (35.6)	517	114 (12.4)	144
Third	552	157 (28.4)	265	58 (10.5)	74
Fourth or later	1018	244 (24.0)	358	73 (7.2)	93
Other	77	16 (20.8)	25	5 (6.5)	5
Unknown or data missing	4	4 (100.0)	6	0	0
Medical history of ILD					
No	2478	713 (28.8)	1116	234 (9.4)	294
Yes	87	31 (35.6)	49	16 (18.4)^*^	22
Unknown or data missing	5	4 (80.0)	6	0	0
Medical history of autoimmune disease					
No	2536	735 (29.0)	1151	248 (9.8)	314
Yes	27	9 (33.3)	14	2 (7.4)	2
Unknown or data missing	7	4 (57.1)	6	0	0
Concomitant ILD					
No	2426	675 (27.8)	1068	215 (8.9)	275
Yes	138	68 (49.3)	96	34 (24.6)^*^^*^	40
Unknown or data missing	6	5 (83.3)	7	1 (16.7)	1
Concomitant autoimmune disease					
No	2465	710 (28.8)	1109	241 (9.8)	305
Yes	98	32 (32.3)	52	8 (8.2)	10
Unknown or data missing	7	6 (85.7)	10	1 (14.3)	1
Medical history of, or concurrent, HBV infection					
No	2525	736 (29.2)	1156	246 (9.7)	311
Yes	27	5 (18.5)	5	3 (11.1)	3
Unknown or data missing	18	7 (38.9)	10	1 (5.6)	2
Previous immunotherapy					
No	1739	543 (31.2)	871	178 (10.2)	228
Yes	826	201 (24.3)	294	72 (8.7)	88
Unknown or data missing	5	4 (80.0)	6	0	0
Type of previous immunotherapy					
Nivolumab	610	143 (23.4)	210	47 (7.7)	60
Pembrolizumab	252	65 (25.8)	96	25 (9.9)	28
Atezolizumab	59	13 (22.0)	16	4 (6.8)	4
Other	3	0	0	0	0
Chemotherapy within previous 3 months					
No	798	251 (31.5)	393	80 (10.0)	97
Yes	1767	492 (27.8)	771	169 (9.6)	218
Unknown or data missing	5	5 (100.0)	7	1 (20.0)	1
EGFR TKI treatment within 3 months prior to the start of atezolizumab treatment					
No	2438	719 (29.5)	1129	242 (9.9)	307
Yes	127	24 (18.9)	35	7 (5.5)	8
Unknown or data missing	5	5 (100.0)	7	1 (20.0)	1
Previous radiotherapy					
No	2197	627 (28.5)	970	211 (9.6)	266
Yes	368	116 (31.5)	194	38 (10.3)	49
Unknown or data missing	5	5 (100.0)	7	1 (20.0)	1
Site of radiotherapy					
Brain	150	48 (32.0)	98	17 (11.3)	23
Bone	129	38 (29.5)	43	11 (8.5)	12
Lungs including lymph nodes	76	21 (27.6)	39	8 (10.5)	11
Other	47	15 (31.9)	22	5 (10.6)	6

^*^ *P* = 0.0056 vs. patients without a history of ILD; ^*^^*^P < 0.0001 vs patients without concurrent ILD.

ECOG, Eastern Cooperative Oncology Group; EGFR TKI, epidermal growth factor receptor tyrosine kinase inhibitor; HBV, hepatitis B virus; ILD, interstitial lung disease.

### Adverse drug reactions of interest

The occurrence of the ADRs of interest is shown in Table 3. The events reported by ≥10 patients were ILD (including MedDRA PT of ILD, pneumonitis, pulmonary fibrosis, and radiation pneumonitis) in 113 patients (4.4%), endocrine disorder (thyroid, adrenal or pituitary dysfunction) in 111 patients (4.3%), hepatic dysfunction in 72 patients (2.8%), colitis and severe diarrhoea in 24 patients (0.9%), encephalitis and meningitis in 18 patients (0.7%), neuropathic disorder in 15 patients (0.6%), severe skin disorder in 15 patients (0.6%), and infusion reactions in 14 patients (0.5%).

**Table 3:** Incidence of adverse drug reactions (ADRs) of interest during treatment with atezolizumab (*n* = 2570)

	Any grade		Grade ≥ 3		Grade 5	
	No. of patients with events (%)	No. of events	No. of patients with events (%)	No. of events	No. of patients with events (%)	No. of events
Any ADR	748 (29.1)	1171	250 (9.7)	316	35 (1.4)	36
ADRs of interest						
ILD	113 (4.4)	115	63 (2.5)	64	21 (0.8)	21
Hepatic dysfunction	72 (2.8)	89	33 (1.3)	39	3 (0.1)	3
Colitis/severe^a^ diarrhoea	24 (0.9)	24	18 (0.7)	18	1 (0.04)	1
Pancreatitis	4 (0.2)	5	2 (0.1)	3	–	–
Type 1 diabetes	3 (0.1)	3	3 (0.1)	3	–	–
Endocrine disorder	111 (4.3)	119	11 (0.4)	11	–	–
Encephalitis or meningitis	18 (0.7)	19	16 (0.6)	17	3 (0.1)	3
Neuropathic disorder	15 (0.6)	15	2 (0.1)	2	–	–
Myasthenia gravis	2 (0.1)	2	–	–	–	–
Severe^a^ skin disorder	15 (0.6)	16	15 (0.6)	16	–	–
Renal dysfunction	7 (0.3)	7	1 (0.03)	1	–	–
Myositis or rhabdomyolysis	4 (0.2)	5	4 (0.2)	5	–	–
Myocarditis	1 (0.03)	1	1 (0.03)	1	–	–
Hemolytic anaemia	1 (0.03)	1	1 (0.03)	1	–	–
ITP	3 (0.1)	3	3 (0.1)	3	–	–
Infusion reaction	14 (0.5)	15	4 (0.2)	4	–	–

ILD, interstitial lung disease; ITP, immune thrombocytic purpura.

^a^Event of Grade ≥ 3 severity.

The incidence of ILD was 4.4% (*n* = 93/2099) in patients with ECOG performance status ≤1 and 4.1% (*n* = 19/465) in those with performance status ≥2 (Table 4). The incidence of ILD was significantly higher in patients with versus without a history of ILD (11.5% vs. 4.2%; *P* = 0.0010) and in those with or without concurrent ILD at baseline (17.4% vs. 3.6%; *P* < 0.0001).

**Table 4:** Incidence of interstitial lung disease in patient subgroups

Patient group	n	No. of patients with ILD (%)	No. of ILD events
Sex			
Male	1797	94 (5.2)	95
Female	773	19 (2.5)	20
Age categories			
<75 years	1912	80 (4.2)	82
≥75 years	658	33 (5.0)	33
Tumour histology			
Adenocarcinoma	1920	83 (4.3)	83
Squamous cell carcinoma	440	20 (4.5)	20
Large cell carcinoma	40	2 (5.0)	2
Other	121	6 (5.0)	8
Unknown or data missing	49	2 (4.1)	2
ECOG performance status			
≤1	2099	93 (4.4)	95
2	387	14 (3.6)	14
3	72	5 (6.9)	5
4	6	0	0
Unknown or data missing	6	1 (16.7)	1
Line of atezolizumab therapy			
Second	919	63 (6.9)	64
Third	552	24 (4.3)	25
Fourth or later	1018	24 (2.4)	24
Other	77	2 (2.6)	2
Unknown or data missing	4	0	0
Medical history of ILD			
No	2478	103 (4.2)	105
Yes	87	10 (11.5)^*^	10
Unknown or data missing	5	0	0
Medical history of autoimmune disease			
No	2536	112 (4.4)	114
Yes	27	1 (3.7)	1
Unknown or data missing	7	0	0
Concomitant ILD			
No	2426	88 (3.6)	90
Yes	138	24 (17.4)^*^^*^	24
Unknown or data missing	6	1 (16.7)	1
Concomitant autoimmune disease			
No	2465	110 (4.5)	112
Yes	98	2 (2.0)	2
Unknown or data missing	7	1 (14.3)	1
Previous immunotherapy			
No	1739	92 (5.3)	94
Yes	826	21 (2.5)	21
Unknown or data missing	5	0	0
Nivolumab	610	10 (1.6)	10
Pembrolizumab	252	10 (4.0)	10
Atezolizumab	59	2 (3.4)	2
Other	3	0	0
Chemotherapy within previous 3 months			
No	798	32 (4.0)	32
Yes	1767	81 (4.6)	83
Unknown or data missing	5	0	0
EGFR TKI treatment within previous 3 months prior to the start of atezolizumab treatment			
No	2438	112 (4.6)	114
Yes	127	1 (0.8)	1
Unknown or data missing	5	0	0
Previous radiotherapy			
No	2197	88 (4.0)	89
Yes	368	25 (6.8)	26
Unknown or data missing	5	0	0
Site of radiotherapy			
Brain	150	6 (4.0)	6
Bone	129	12 (9.3)	12
Lungs including lymph nodes	76	10 (13.2)	11
Other	47	2 (4.3)	2

ECOG, Eastern Cooperative Oncology Group; EGFR TKI, epidermal growth factor receptor tyrosine kinase inhibitor; ILD, interstitial lung disease.

^*^ *P* = 0.0010 vs. patients without a history of ILD; ^*^^*^P < 0.0001 vs. patients without concurrent ILD.

**Figure 2:**
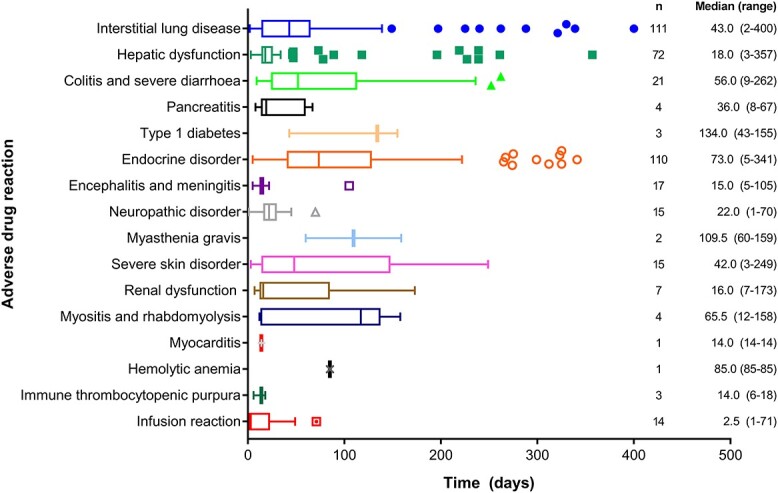
Box and whisker plot showing the time to onset of immune-related adverse drug reactions. The box shows the interquartile range (IQR), the vertical line in each box is the median and horizontal bars indicate the 25th and 75th percentile plus 1.5 times IQR or the range (maximum to minimum) if the values in the range fall within the 25th and 75th percentile plus 1.5 times IQR. Data points outside of the 25th and 75th percentile plus 1.5 times IQR are plotted as outliers.

The median time to onset of the ADRs of interest that occurred in ≥10 patients are shown in Figure 2. Some had a median onset of <25 days, including infusion reactions (median 2.5 days), encephalitis/meningitis (median 15.0 days), hepatic dysfunction (median 18.0 days), and neuropathic disorder (median 22.0 days), whereas others were slower to develop, including severe skin disorder (median 42.0 days), ILD (median 43.0 days), colitis/severe diarrhoea (median 56.0 days) and endocrinopathy (73.0 days).

Supplementary Table S1 shows how the ADRs of interest were managed. Atezolizumab was discontinued in 95/115 occurrences of ILD (82.6%), 15/19 occurrences of encephalitis/meningitis (78.9%) and 11/16 occurrences of severe skin disorders (68.8%). However, few infusion reactions (4/15; 26.7%) or endocrine disorders (15/119; 12.6%) led to atezolizumab discontinuation.

### Outcomes

The number of cases of recovery/remission and the number of days from onset to recovery/remission are shown in Supplementary Table S2. Thirty-five patients died as a result of 36 ADRs, in which a causal relationship to atezolizumab could not be ruled out, including 20 deaths from ILD, two each from meningitis, lung disorder, and liver disorder, and one each from hemophagocytic lymphohistiocytosis, immune-mediated encephalitis, myocardial infarction, non-cardiogenic pulmonary edema, pneumonitis, colitis, hepatobiliary disease, pneumonia bacterial, pulmonary embolism, and death (by PT). Among the 35 fatal cases, the most common concurrent conditions were ILD (*n* = 11) and diabetes mellitus (*n* = 7); 13 patients who died had received radiotherapy within 3 months before the start of treatment with atezolizumab. There were no characteristics such as age, sex, histology, PS, treatment line, or prior treatment that were predictive of a Grade 5 ADR (data not shown).

## Discussion

This large-scale post-marketing surveillance study confirms that the tolerability and safety of atezolizumab in a heterogeneous real-world population of Japanese patients with NSCLC is comparable to the profile established in the randomized phase III clinical trials. No new safety signals were identified and the risk and onset of immune-related AEs were as expected.

Immune-related AEs are expected in patients receiving immunotherapy, and may in fact be a marker of treatment response (15). An analysis of outcomes in Japanese NSCLC patients receiving the PD-1 inhibitor nivolumab found that the development of immune-related AEs was a significant predictor of OS (15). Most of the immune-related AEs develop within the first 3 months of starting of immunotherapy, as was seen in our study. Median time to the onset of skin, endocrine, gastrointestinal and infusion-related immune-related AEs with atezolizumab in the current analysis was similar to the median onset of these events with nivolumab reported in the literature (16). In our study, the onset of hepatic (median 18 days), pulmonary (median 43 days), and renal (median 16 days) immune-related AEs was earlier than reports of these events with nivolumab (16), but the range of onset was wide with both agents, and no comparative data are available, so these findings should be considered hypothesis-generating.

It has been reported that the incidence of Grade ≥ 3 AEs with immune checkpoint inhibitors is similar in patients aged <75 and ≥75 years old (17,18). The results of the current study are consistent with these findings; the incidence of Grade ≥ 3 ADRs was exactly the same (9.7%) in patients aged <75 years and ≥75 years, confirming that patients aged ≥75 years are not at increased risk of developing a Grade ≥ 3 ADR during atezolizumab treatment.

Studies with the PD-1 inhibitor nivolumab have reported an increase in the incidence of severe pneumonia in patients with poor performance status (19). However, in our study, the incidence of ILD was 4.4% in patients with performance status of ≤1 and 4.1% in those with performance status ≥2, indicating no higher risk with worsening performance status during atezolizumab treatment.

The incidence of Grade ≥ 3 ADRs by treatment line was 12.4% in patients receiving atezolizumab as second-line treatment, 10.5% as third-line treatment and 7.2% as fourth-line or later lines of treatment, showing no effect of treatment line on the risk of ADRs during atezolizumab. This is generally true of immunotherapy, where studies have shown a comparable incidence of AEs, regardless of the line of therapy, in patients who are receiving immunotherapy or targeted agents (16,20), but differs from the situation with chemotherapy, in which patients frequently experience cumulative toxicity and worsening tolerability as they proceed through multiple lines of treatment (21).

During the use of immune checkpoint inhibitors for NSCLC, attention should be paid to the development of ILD (22). As described earlier, the anti-PD-L1 antibodies, which have little effect on PD-L2 (8), are associated with a lower incidence of pneumonitis compared with the anti-PD-1 antibodies (9). The incidence of ILD in our study was 4.4%, which is higher than the incidence in the Japanese subgroup of patients in the phase III OAK study (1.8%) (23), but lower than in a previous report with the PD-1 inhibitor pembrolizumab in Japanese patients with NSCLC (22.2%) (24). These differences likely reflect the proportion of patients in each study with underlying lung disease. For example, the OAK study specifically excluded patients with a history of idiopathic pulmonary fibrosis (including pneumonitis), drug-induced pneumonitis, organizing pneumonia or active pneumonitis (10). In contrast, 13.9% of patients had pre-existing ILD at baseline in the pembrolizumab study, whereas in our study, 3.4% of patients had a history of ILD and 5.4% had ILD at baseline. We noted that the incidence of ILD during atezolizumab treatment was 4.2% in the group without a history of ILD compared with 11.5% in the group with a history of ILD (*P* = 0.0010), and 3.6% in the group without a complication of ILD compared with 17.4% in the group with a complication of ILD (*P* < 0.0001). It should also be noted that the incidence of ILD is higher in Japan (4.0%) than in the rest of the world (0.2%) (25). These data suggest that Japanese physicians should be alert to an increased risk of ILD development during atezolizumab treatment, particularly in patients with a history of, or concurrent, ILD, and monitor these patients closely.

There are concerns about an increased risk of immune-related AEs or worsening of autoimmune diseases when immune checkpoint inhibitors are used in patients with concurrent autoimmune diseases (26). However, in this report, the incidence of Grade ≥ 3 ADRs was 9.8% in patients without concurrent autoimmune disease and 8.2% in patients with concurrent autoimmune diseases.

Strengths of our study were the large patient cohort of more than 2500 Japanese patients with NSCLC, including many patients aged ≥75 years, with a history of or concurrent ILD, and with a PS ≥2, who were followed up for ≥1 year. However, the study limitations were the single-arm design with no control group, and the fact that we did not evaluate the effectiveness of atezolizumab. However, based on the large number of patients and their varied characteristics, we can be confident that our data provide a comprehensive assessment of the tolerability and safety of atezolizumab in clinical practice in Japan. These data can be used as the basis for future research, including an assessment of the effectiveness of atezolizumab.

## Conclusion

A large, multicenter, prospective, clinical cohort study of >2500 patients confirmed the excellent clinical tolerability of atezolizumab in a real-world population of Japanese patients with NSCLC. Careful monitoring is considered necessary in patients with a past/current history of ILD, because the rate of fatal ILD was higher in these patients than in those without such a history.

## Supplementary Material

Atezolizumab_PMS_for_submission_supplementary_material_hyac024
