# Supplementary material for: The real-world safety of atezolizumab as second-line or later treatment in Japanese patients with non-small-cell lung cancer: a post-marketing surveillance study
Source: Jpn J Clin Oncol. 2022 Mar 23;52(6):623–32. doi: 10.1093/jjco/hyac024 (PMC9157296; doi:10.1093/jjco/hyac024)
Supplement: Atezolizumab_PMS_for_submission_supplementary_material_hyac024 [file atezolizumab_pms_for_submission_supplementary_material_hyac024.docx]

***The Real-World Safety of Atezolizumab as Second-Line or Later Treatment
 in Japanese Patients with Non-Small-Cell Lung Cancer:
A Post-Marketing Surveillance Study***

**Authors**

Yuichiro Ohe,^1^ Naoya Yamazaki,^2^ Nobuyuki Yamamoto,^3^ Haruyasu Murakami,^4^ Kiyotaka Yoh,^5^ Shigehisa Kitano,^6^ Hideyuki Hashimoto,^7^ Ayako Murayama,^7^ Sayuri Nakane,^7^ Akihiko Gemma^8^

**Affiliations**

**1** Department of Thoracic Oncology, National Cancer Center Hospital, Tokyo, Japan

**2** Department of Dermatologic Oncology, National Cancer Center Hospital, Tokyo, Japan

**3** Internal Medicine III, Wakayama Medical University, Wakayama, Japan

**4** Division of Thoracic Oncology, Shizuoka Cancer Center, Sunto-gun, Japan

**5** Department of Thoracic Oncology, National Cancer Center Hospital East, Kashiwa, Japan

**6** Division of Cancer Immunotherapy Development, Advanced Medical Development Center, The Cancer Institute Hospital of the Japanese Foundation for Cancer Research, Tokyo, Japan

**7** Real World Data Science Department, Drug Safety Division, Chugai Pharmaceutical Co., Ltd, Tokyo, Japan

**8** Department of Pulmonary Medicine and Oncology, Graduate School of Medicine, Nippon Medical School, Tokyo, Japan

**Corresponding author**

Dr Yuichiro Ohe, National Cancer Center Hospital, 5-1-1 Tsukiji, Chuo-ku, Tokyo, 104-0045 Japan. Tel: +81-3-3542-2511; Fax: +81-3-3545-5370. Email: yohe@ncc.go.jp
**Running head:** Real-world safety of atezolizumab in NSCLC

# Supplementary Material

**Supplementary Table S1.** Adverse drug reactions of interest requiring treatment interruption or steroids

|  |  | **No. of events requiring treatment interruption (%)^a^** | | | **No. of events treated with steroids (%)^b^** | |
| --- | --- | --- | --- | --- | --- | --- |
|  | **No. of patients with ADRs (events)** | **No disruption** | **Temporary discontinuation** | **Permanent discontinuation** | **No steroid treatment** | **Steroids required** |
| All ADRs | 748 (1,171) | 534 (45.6) | 191 (16.3) | 441 (37.7) | 820 (70.0) | 343 (29.3) |
| ADRs of interest |  |  |  |  |  |  |
| ILD | 113 (115) | 8 (7.0) | 12 (10.4) | 95 (82.6) | 19 (16.5) | 95 (82.6) |
| Hepatic dysfunction | 72 (89) | 26 (29.2) | 15 (16.9) | 48 (53.9) | 64 (71.9) | 25 (28.1) |
| Colitis and severe^c^ diarrhea | 24 (24) | 3 (12.5) | 8 (33.3) | 13 (54.2) | 3 (12.5) | 21 (87.5) |
| Pancreatitis | 4 (5) | 2 (40.0) | 1 (20.0) | 2 (40.0) | 5 (100.0) | 0 |
| Type 1 diabetes | 3 (3) | 0 | 1 (33.3) | 2 (66.7) | 3 (100.0) | 0 |
| Endocrine disorder | 111 (119) | 73 (61.3) | 30 (25.2) | 15 (12.6) | 97 (81.5) | 21 (17.6) |
| Encephalitis and meningitis | 18 (19) | 1 (5.3) | 3 (15.8) | 15 (78.9) | 1 (5.3) | 18 (94.7) |
| Neuropathic disorder | 15 (15) | 7 (46.7) | 2 (13.3) | 6 (40.0) | 9 (60.0) | 5 (33.3) |
| Myasthenia gravis | 2 (2) | 0 | 0 | 2 (100.0) | 0 | 2 (100.0) |
| Severe^c^ skin disorder | 15 (16) | 2 (12.5) | 3 (18.8) | 11 (68.8) | 4 (25.0) | 12 (75.0) |
| Renal dysfunction | 7 (7) | 2 (28.6) | 1 (14.3) | 4 (57.1) | 5 (71.4) | 2 (28.6) |
| Myositis and rhabdomyolysis | 4 (5) | 0 | 0 | 5 (100.0) | 0 | 5 (100.0) |
| Myocarditis | 1 (1) | 0 | 0 | 1 (100.0) | 0 | 1 (100.0) |
| Hemolytic anemia | 1 (1) | 0 | 0 | 1 (100.0) | 0 | 1 (100.0) |
| ITP | 3 (3) | 0 | 0 | 3 (100.0) | 0 | 3 (100.0) |
| Infusion reaction | 14 (15) | 11 (73.3) | 0 | 4 (26.7) | 9 (60.0) | 6 (40.0) |

ADR, adverse drug reaction; ILD, interstitial lung disease; ITP, immune thrombocytic purpura.

^a^Data are not available in 5 patients for all ADRs and in 1 patient with immune-related ADRs (endocrine disorder); ^b^Data are not available in 8 patients for all ADRs, in 3 patients with immune-related ADRs (1 patient each for ILD, endocrine disorders, and neuropathic disorders); ^c^Event of Grade ≥3 severity.

**Supplementary Table S2.** Time to recovery or remission from adverse drug reactions of interest

|  |  |  | **Time to recovery or remission (days)** | | |
| --- | --- | --- | --- | --- | --- |
|  | **No. of patients with ADRs (events)** | **Recovery or remission events** | **N** | **Median** | **Range** |
| All ADRs | 748 (1,171) | 944 | 939 | 22.0 | 1–487 |
| ADRs of interest |  |  |  |  |  |
| ILD | 113 (115) | 68 | 67 | 42.0 | 9–287 |
| Hepatic dysfunction | 72 (89) | 76 | 76 | 27.5 | 4–321 |
| Colitis and severe diarrhea | 24 (24) | 20 | 19 | 38.0 | 7–387 |
| Pancreatitis | 4 (5) | 4 | 4 | 44.0 | 18–81 |
| Type 1 diabetes | 3 (3) | 3 | 3 | 57.0 | 10–309 |
| Endocrine disorder | 111 (119) | 80 | 80 | 43.0 | 1–383 |
| Encephalitis and meningitis | 18 (19) | 15 | 15 | 21.0 | 4–97 |
| Neuropathic disorder | 15 (15) | 6 | 6 | 36.0 | 4–84 |
| Myasthenia gravis | 2 (2) | 2 | 2 | 75.5 | 36–115 |
| Severe skin disorder | 15 (16) | 14 | 14 | 35.5 | 8–211 |
| Renal dysfunction | 7 (7) | 5 | 5 | 12.0 | 4–106 |
| Myositis and rhabdomyolysis | 4 (5) | 2 | 2 | 84.5 | 36–133 |
| Myocarditis | 1 (1) | 1 | 1 | 16.0 | 16–16 |
| Hemolytic anemia | 1 (1) | 1 | 1 | 14.0 | 14–14 |
| ITP | 3 (3) | 3 | 3 | 57.0 | 8–182 |
| Infusion reaction | 14 (15) | 15 | 15 | 2.0 | 1–368 |

ADR, adverse drug reaction; ILD, interstitial lung disease; ITP, immune thrombocytic purpura.
